# Test-retest reliability and sensitivity of senior elite amateur boxers maximal punch force, as quantified by a vertically mounted force plate

**DOI:** 10.1371/journal.pone.0289791

**Published:** 2023-08-10

**Authors:** Mitchell James Finlay, Richard Michael Page, Matt Greig, Craig Alan Bridge

**Affiliations:** 1 Sports Performance Research Group, Department of Sport & Physical Activity, Edge Hill University, Ormskirk, Lancashire, United Kingdom; 2 Sport Department, University Academy 92 (UA92), Manchester, United Kingdom; Sheffield Hallam University, UNITED KINGDOM

## Abstract

An ecologically valid, reliable and sensitive method of quantifying punch force variables would be useful for coaches and practitioners monitoring combat-specific performance. The present study utilised a vertically mounted force plate to quantify the peak punch force and rate of force development (RFD) of amateur boxers. Ten male senior elite amateur boxers performed maximal jab, cross, and hook punches across two separate days. The force plate showed excellent within-day and good-to-excellent between-day reliability for peak punch impact force and RFD (ICC 0.89–0.99). The CV% for all punch force variables were similar on day 1 (3–9%) and day 2 (4–10%). Standard error of measurement (SEM) and smallest worthwhile changes (SWC) revealed the force plate can detect small-to-moderate changes in punch performance. The greatest impact forces and RFD were found in the rear hook (2624 ± 581 N, 296448 ± 101823 N.s^-1^), followed by the lead hook (2524 ± 532 N, 256813 ± 81735 N.s^-1^), cross 2425 ± 545 N, 193004 ± 62671 N.s^-1^) and jab (1645 ± 537 N, 116675 ± 41577 N.s^-1^). The vertically mounted force plate is a reliable and sensitive test of punch performance, thus may be useful in determining the efficacy of training interventions.

## Introduction

For the coach or practitioner working in combat striking sports, the quantification of punch peak force and rate of force development (RFD) (the rate at which combat athletes can produce force) may be key performance indicators (KPI’s) [1–3], thus, potentially justifying or influencing sport-specific training or strength and conditioning practices through the observation of effective changes in performance [1, 2]. However, the task of directly monitoring punch force variables has traditionally been challenging for scientists, consequently, a range of methodological approaches have been adopted in the literature. Researchers have embedded or attached accelerometers to punch bags, punch balls, mannequins and ballistic pendulums [4–8], at times using mathematical modelling to estimate punch force from the accelerations of the target. This indirect method requires the collection and subsequent analysis of several kinetic and kinematic variables, with sampling frequency, accuracy, and reliability data frequently not reported. Further, this method often requires the conversion of data derived from the software to SI units for analysis [6]. Pierce and colleagues [9] utilised an innovative and direct method of embedding force sensors inside a glove to measure punch force, though the validity of the sensor in a punching action was not reported. Indeed, whilst many of the above methods add useful insights into punching performance of combat athletes, they would potentially under-or-over estimate punch force when compared to direct measurement.

The measurement of punch force, as with any performance measure, must be ecologically valid and reliable for it to be effective, and must also be sensitive enough to detect changes in performance [10–13]. Test-retest reliability concerns the reproducibility of the observed value when the measurement is repeated [10, 12, 13]. Hopkins states that greater reliability implies better precision of single measurements and better tracking of changes in measurement. The use of force plates, load cells, and/or dynamometers with piezoelectrical force transducers may be the gold standard in measuring human impact forces. Regarding the direct measurement of punch impact forces, Smith and colleagues initially proposed the use of such equipment in amateur boxing, having published a series of pioneering intervention work in the area [14–17]. In recent years, researchers in Australia have utilised a wall‐mounted S‐beam load cell with a cushioned target, alternatively named a ‘punching integrator’ [24, 25]. The authors reported very good mechanical reliability and accuracy (error <0.1%), assessed by calculating the typical error (TE) and coefficient of variation (CV) for a range of known masses [1, 18]. Dunn and colleagues [1] reported moderate-to-high within-day reliability ICC 0.85 (0.67–0.94) in peak impact force when all punch types were considered, though a learning effect on day 2 testing was observed.

A wall-mounted structure; however, may not accommodate certain punch types such as hooks and uppercuts, reflecting in many studies only analysing straight punches. The punching integrator used by Dunn et al., [1] and Halperin et al., [18] overcame this issue by having the target protrude from the wall. This allowed the participants to step to the sides and deliver hooks at an angle, reflecting the way a boxer may switch angles during a counter, or during combination punching. Future development of vertically mounted devices could be designed in such a way that it protrudes from a solid structure, thus enabling the quantification of hook punch forces. Likewise, any future instrument should also allow for changes in the height of the punching target relative to individual participants stature. Both of the above would, in turn, increase the ecological validity of the instrument.

The considerable impact forces associated with punches in amateur boxing was highlighted earlier, and the potential for injury when striking a mounted target must also be considered. Therefore, to reduce impact forces, thus reducing the likelihood of injury, any vertically mounted instrument typically comprises protective padding [1, 3, 15–18] in addition to the standard application of hand wraps and gloves to boxers’ hands. It is worth noting that this force attenuation of the protective padding, would inevitably result in a less accurate reading of impact force. However, this may not be problematic for the practitioner, providing the assessment is ecologically valid, reliable, and sensitive to changes in performance [19, 20].

The development of a reliable, and sensitive method of assessing punch force variables, that is ecologically valid, may allow for the monitoring of punch performance, or in assessing the efficacy of acute and longer-term training interventions. Therefore, the main aim of this study was to examine the test-retest reliability and sensitivity of amateur boxer’s punch force variables, as quantified by a vertically mounted force plate. A secondary aim of the study was to analyse and report the punch force capabilities of senior elite amateur boxers.

## Methods

### Participants

Ten male senior elite amateur boxers (age 19.7 ± 1.2 years; stature 180.9 ± 7.0 cm; body mass 78.7 ± 9.6 kg) volunteered to take part in the study. As strict inclusion criteria for participation in the current study, boxers were defined as senior elite if they had previously competed at the National Elite Championships of their respective nation, in accordance with the classification from England Boxing [21]. Boxers were initially contacted via social media platforms, email, and through in person meetings with themselves or their coaches, at boxing gyms in the North of England. Thus, the participants were chosen via purposive sampling. All boxers provided written informed consent and were informed of the risks and benefits before participation. Trained and experienced boxers were chosen to minimise the variability in repetitions of a specific movement [22]. Ethical approval for this study was provided by Edge Hill University’s Subject Research Ethics Committee (SREC) (ETH2021-0058) and was conducted in accordance with the Helsinki Declaration (7^th^ Revision). All testing took place within the England amateur boxing season (September–May).

### Procedures

#### Experimental overview

Participants were required to attend the laboratory on two separate days, no more than 48hrs apart. Across the two days, three separate trials (Day 1 AM and PM; Day 2 PM) of maximal punch testing against a vertically mounted force plate was conducted. This experimental design allowed for the completion of the primary aim, to analyse the test re-test (within-day and between-day) reliability and sensitivity of punch force variables, and to report the punch force capabilities of senior elite amateur boxers. Prior to these sessions, the participants took part in a familiarisation session, whereby they could experience the simulation protocol discussed later, and punching the force plate at both sub-maximal and maximal intensities. The maximal punching protocol included the jab, cross, and lead and rear hooks. The maximal punches were performed at the end of a standardised 15-minute warm-up, detailed in the next section.

#### Experimental protocol

**Fig 1:**
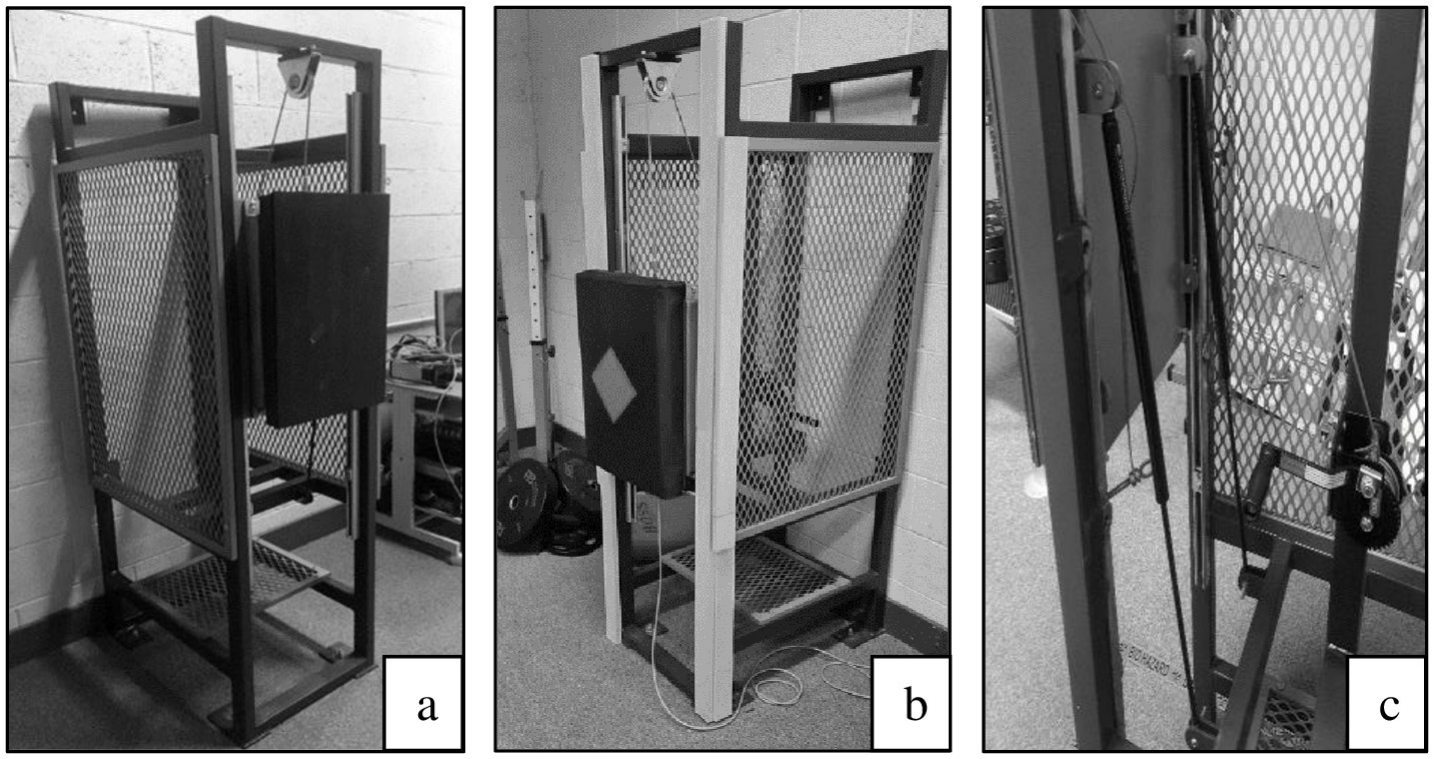
a) Force plate and structure b) Force plate and structure with protective padding c) Winch ratchet mechanism, gas struts and bolts supporting the mass of the force plate.

During each testing session, the participants performed two maximal effort jab, cross, lead hook, and rear hook punches to the vertically mounted force plate, with each punch interspersed by a 5-second rest. The jab is a straight punch from the lead hand that moves along the sagittal plane [23] and is primarily used to set-up a combination [1] of more damaging punches, or to manage distance. The cross, or otherwise termed a rear hand, is a straight punch thrown to inflict damage, and as the name suggests, is thrown from the rear hand [23]. Thomson and colleagues describe the lead and rear hook punches as a sweeping motion that moves along the transverse plane, and these punches are also thrown to inflict damage [23]. Before the maximal punching protocol, the participants initially performed a standardised 3-minute round of shadow boxing, dynamic activation, and mobility exercises, and a single 3-minute round of the Boxing-specific Exercise Protocol (BSEP) [24, 25] on a punch bag. Inclusion of task-specific activity in the warm-up is recommended to ensure ecological validity [26]. Before, and at every 1-minute interval of the warm-up BSEP round, participants performed sub-maximal efforts of the punch types described earlier, at perceived progressive intensities (50%, 70%, 90%, and 100%) [1] to the force plate. The sub-maximal punches comprised the same procedures as the maximal punch protocol. In both maximal and sub-maximal punching tasks, the participants were asked to strike a red diamond target located on a foam padding case (Fig 1b), corresponding to the middle of the force plate, details of which are presented in the subsequent section. Additionally, participants were asked to perform all punches at a self-selected distance to replicate punching technique in training and competition [3], thus increasing the ecological validity. Finally, boxers were asked to wear their own hand-wraps and 12 oz gloves with velcro strap throughout each testing session. Gloves were supplied to participants if their own gloves did not conform to the above.

#### Experimental measures

A force plate (Bertec, USA) sampling at 2000 Hz was used to quantify punch force variables, specifically, peak punch force and RFD (the maximum amount of force/RFD produced during a punch). Relative punch force was also calculated as peak force divided by body mass. The force plate was vertically mounted to a custom-built steel apparatus 200 x 65 x 82cm and bolted to the ground and wall (Fig 1a). The structure protruded from the wall in such a way as to allow hook punches to be performed. The force plate was bolted to a large metal back plate attached to the steel apparatus and comprised three safety mechanisms. Specifically, the mass of the force plate was accommodated by bolts, a winch ratchet mechanism, and two gas struts (Fig 1c), which together with the drilling of several bolt holes, enabled the positioning of the punching target to be adjusted specific to individual boxer stature. To attenuate the impact forces produced in maximal punches, thus protecting the boxers from injury, custom foam padding was attached to the force plate and the surrounding steel structure (Fig 1b). The padding consisted of high-density foam (72 x 42 x 10cm) replicating material typically found in wall-mounted targets in boxing gyms, and this was enclosed in a rectangular case with a clear punching target stitched into the middle.

Force data was captured using a motion capture system (Qualysis, Sweden). Specifically, force signals were transferred to a AM6500 digital signal converter. Raw vertical force data was exported to Visual 3D software, whereby a pipeline command identified the beginning and end of each punch, with a minimum threshold of 200 N. All equipment utilised was risk assessed and calibrated in accordance with the specific manufacturer guidelines prior to data collection.

### Statistical analyses

Statistical analyses and the variables that were to be included were decided a priori. The normality of data distribution was initially assessed via the Shapiro-wilks test, and potential outliers were assessed via the inspection of box plots. Systematic bias was explored via a paired samples t-test [27], whereby differences in absolute and relative (to body mass) peak impact force, and RFD between trials, could be explored. Specifically. within-day reliability related to differences between trial 1 (AM) and trial 2 (PM) on day 1 only. Likewise, a paired samples t-test was used to assess between-day reliability, related differences in the average mean of all trials on day 1, and the PM trial on day 2. Relative reliability was determined by calculating the Intraclass correlation coefficient (ICC), whereby the following thresholds were applied: < 0.5 poor, 0.50–0.75 moderate, 0.75–0.90 good and > 0.90 excellent [28]. The ICC can be defined as follows: between-subjects variability ÷ (between-subjects variability + error); as the error term decreases the ICC moves from 0 to 1 indicating perfect reliability [10]. In addition to relative reliability, absolute reliability was assessed via the standard error of measurement (SEM) and coefficient of variation (CV%) (calculated as SD/mean x 100), whilst 95% limits of agreement were also reported. The smallest worthwhile change (SWC) was calculated based on the between subject SD for small (0.2), moderate (0.6), and large (1.2) effect sizes, and compared to the SEM values [1, 29]. Comparison of the SWC and SEM was performed in order to determine the test sensitivity in detecting systematic variation in performance [12, 29]. Pearson correlation analysis was used to explore the relationships between all punch force variables. All statistical analyses were performed using an adapted Microsoft Excel sheet [12] and SPSS v.25 (IBM Inc., Chicago, IL), with statistical significance assumed at P ≤ 0.05.

## Results

### Within-day and between-day reliability, and sensitivity

As identified in Table 1, absolute peak punch force and RFD had excellent within-day (ICC 0.96–0.99; 0.93–0.97) and good to excellent between-day reliability (ICC 0.89–0.98; 0.94–0.97). Likewise, within-day relative peak punch force was good to excellent (ICC 0.89–0.95), whilst between-day relative peak punch impact force was moderate to good (ICC 0.58–0.95). Significant within-day differences in absolute and relative peak punch force were found in the cross (121 N; *t*(9) = 3.008, *p* = 0.015,; 1.5 N.kg^-1^, *t*(9) = 3.047, *p* = 0.014) and rear hook (87 N, *t*(9) = 2.951, *p* = 0.016,; 1.2 N.kg^-1^,*t*(9) = 2.689, *p* = 0.025). There was also a significant within-day difference in the relative peak punch force of the jab (2.0 N.kg^-^1, *t*(9) = 3.672, *p* = 0.021). No other significant within-day differences, and indeed no between-day differences were observed in absolute or relative punch force, and RFD across all punches. The CV% for all variables were similar on day 1 (3–9%) and day 2 (4–10%), though as can be seen in Table 1, this varied between punch variables and punch type. Similarly, regarding sensitivity, Table 1 shows the SEM and SWC data for each punch type and punch variable. For most punch variables, the SEM was similar to, or slightly greater than the SWC _(0.2)_ but smaller than the SWC _(0.6)_, with the exception of absolute peak force in the cross and rear hook on day 1, and jab and lead hook on day 2 (< SWC _0.2_). Likewise, when comparing the average values of day 1 with the average of day 2, SEM of peak relative force in the lead and rear hook, was greater than SWC _(0.6)_, but smaller than SWC _(1.2)_.

**Table 1:** Within-day and between-day reliability of punch force variables obtained from maximal intensity punches against the vertically mounted force plate.

**Day 1**									
Punch	Metric	Test 1	Test 2	Difference	95% LOA	ICC (95% CI)	CV%	SEM (95% CI)	SWC _(0.2, 0.6, 1.2)_
**Jab**	Peak force (N)	1534 ± 514	1699 ± 603	164	383	0.96 (0.83–0.99)	8	138 (95–252)	112, 336, 672
	Relative force (N.kg^-1^)	19.3 ± 4.7	21.3 ± 5.3	2.0*	4.5	0.92 (0.72–0.98)	8	1.6 (1.1–2.9)	1.0, 3.0, 6.0
	RFD (N.s^-1^)	109866 ± 37356	115698 ± 39750	5832	36901	0.93 (0.74–0.98)	9	11930 (8206–21779)	7714, 23143, 46286
**Cross**	Peak force (N)	2328 ± 547	2449 ± 599	121*	249	0.98 (0.930–0.996)	4	90 (62–164)	115, 344, 688
	Relative force (N.kg^-1^)	29.3 ± 3.7	30.8 ± 4.0	1.5*	2.9	0.94 (0.79–0.99)	4	1.1 (0.7–2.0)	0.8, 2.3, 4.7
	RFD (N.s^-1^)	177886 ± 56742	195217 ± 70138	17331	48045	0.95 (0.80–0.99)	6	17334 (11923–31644)	12758, 38275, 76551
**Lead hook**	Peak force (N)	2443 ± 569	2533 ± 576	90	349	0.96 (0.86–0.99)	4	126 (87–230)	114, 343, 687
	Relative force(N.kg^-1^)	30.8 ± 3.9	31.9 ± 4.3	1.2	4.9	0.87 (0.55–0.97)	4	1.7 (1.2–3.1)	0.8, 2.5, 5.0
	RFD (N.s^-1^)	253108 ± 82059	263039 ± 88313	38766	49896	0.97 (0.88–0.99)	7	18974 (13051–34640)	17971, 53912, 107825
**Rear hook**	Peak force (N)	2576 ± 632	2663 ± 562	88 *	184	0.99 (0.97–1.00)	3	67 (46–121)	120, 359, 718
	Relative force (N.kg^-1^)	32.4 ± 4.5	33.6 ± 3.6	1.2*	2.7	0.95 (0.83–0.99)	3	1.0 (0.7–1.9)	0.8, 2.5, 4.9
	RFD (N.s^-1^)	294404 ± 108321	302420 ± 105886	8016	62112	0.97 (0.88–0.99)	7	22408 (15413–40909)	21422, 64266, 128532
**Day 2**									
		Test 1	Test 2	Difference	95% LOA	ICC (95% CI)	CV%	SEM (95% CI)	SWC _(0.2, 0.6, 1.2)_
**Jab**	Peak force (N)	1623 ± 551	1723 ± 541	100	240	0.98 (0.93–1.00)	5	87 (60–158)	109, 328, 655
	Relative force (N.kg^-1^)	20.3 ± 4.7	21.6 ± 4.8	1.4	3.5	0.95 (0.80–0.99)	5	1.3 (0.9–2.3)	0.9, 2.8, 5.7
	RFD (N.s^-1^)	115559 ± 43375	125578 ± 49917	10018	33655	0.95 (0.81–0.99)	5	12141 (8351–22166)	9352, 28056, 56112
**Cross**	Peak force (N)	2410 ± 541	2512–564	102	339	0.96 (0.86–0.99)	4	122 (84–223)	111, 332, 663
	Relative force (N.kg^-1^)	30.4 ± 3.7	31.7 ± 4.0	1.3	4.3	0.88 (0.58–0.97)	4	1.6 (1.1–2.8)	0.8, 2.3, 4.7
	RFD (N.s^-1^)	194869 ± 56986	204044 ± 72352	9176	50231	0.94 (0.79–0.99)	8	18121 (12465–33083)	13025, 39074, 78148
**Lead hook**	Peak force (N)	2525 ± 515	2594 ± 543	70	296	0.97 (0.89–0.99)	4	106 (73–193)	107, 318, 635
	Relative force (N.kg^-1^)	31.9 ± 3.4	32.7 ± 3.6	0.8	3.9	0.87 (0.56–0.97)	4	1.4 (1.0–2.6)	0.7, 2.1, 4.2
	RFD (N.s^-1^)	249817 ± 80440	261289 ± 78717	11473	31174	0.99 (0.94–1.00)	4	11246 (7736–20532)	15917, 47750, 95500
**Rear hook**	Peak force (N)	2603 ± 593	2655 ± 624	53	408	0.96 (0.84–0.99)	4	147 (101–269)	122, 365, 730
	Relative force (N.kg^-1^)	32.8 ± 4.3	33.5 ± 5.2	0.7	5.1	0.88 (0.60–0.970)	4	1.9 (1.3–3.4)	1.0, 2.9, 5.7
	RFD (N.s^-1^)	286656 ± 102027	302314 ± 106696	15657	91648	0.92 (0.73–0.98)	10	33063 (22742–60361)	20878, 62633, 125266
**Between-day**									
Punch		Day 1 average	Day 2 average	Difference	95% LOA	ICC (95% CI)	CV%	SEM (95% CI)	SWC _(0.2, 0.6, 1.2)_
**Jab**	Peak force (N)	1616 ± 552	1673 ± 543	56	249	0.98 (0.92–1.00)	5	90 (62–164)	109, 328, 657
	Relative force (N.kg^-1^)	20.3 ± 4.9	21.0 ± 4.6	0.7	3.3	0.95 (0.83–0.99)	5	1.2 (0.8–2.2)	1.0, 2.9, 5.7
	RFD (N.s^-1^)	112782 ± 37638	120568 ± 45965	7786	33708	0.94 (0.79–0.99)	7	11529 (7930–21047)	8402, 25205, 50410
**Cross**	Peak force (N)	2389 ± 570	2461 ± 546	72	368	0.96 (0.84–0.99)	4	133 (91–242)	112, 335, 670
	Relative force (N.kg^-1^)	30.1 ± 3.8	31.1 ± 3.7	1.0	4.7	0.84 (0.48–0.96)	4	1.7 (1.2–3.1)	0.8, 2.3, 4.5
	RFD (N.s^-1^)	186552 ± 62604	199457 ± 63850	12905	35715	0.97 (0.89–0.99)	6	12568 (8645–22944)	12646, 37938, 75876
**Lead hook**	Peak force (N)	2488 ± 565	2560 ± 524	72	443	0.91 (0.68–0.98)	7	188 (130–344)	109, 327, 654
	Relative force (N.kg^-1^)	31.3 ± 4.0	32.3 ± 3.4	0.9	7.1	0.58 (-0.03–0.88)	7	2.5 (1.7–4.6)	0.7, 2.2, 4.4
	RFD (N.s^-1^)	258073 ± 88847	255553 ± 79185	-2520	59743	0.95 (0.82–0.99)	7	21553 (14825–39348)	16831, 50493, 100986
**Rear hook**	Peak force (N)	2620 ± 596	2629 ± 600	10	615	0.89 (0.63–0.97)	7	222 (152–405)	120, 359, 717
	Relative force (N.kg^-1^)	33.0 ± 4.0	33.1 ± 4.6	0.2	8.0	0.61 (0.01–0.86)	7	2.9 (2.0–5.3)	0.9, 2.6, 5.2
	RFD (N.s^-1^)	298412 ± 105932	294485 ± 101736	-3927	61032	0.97 (0.87–0.99)	6	22019 (15145–40197)	20771, 62313, 124626

ICC = Intraclass correlation coefficient; LOA = Limits of agreement; Newtons; N.kg-1 = Newtons / body mass; N.s = Newtons per second; RFD = Rate of force development; SEM = Standard error of measurement; SWC = Smallest worthwhile change; RFD = Rate of force development; N = Newtons; N.kg-1 = Newtons / body mass; N.s = Newtons per second.

*Significant difference (p ≤ 0.05) between test 1 and test 2 on same day.

### Correlations between punch variables

There was a statistically significant, positive correlation between absolute peak impact force and RFD in the jab (*r*_*s*_(10) = 0.745, *p* = 0.013); cross (*r*_*s*_(10) = 0.895, *p* < 0.0001); lead hook (*r*_*s*_(10) = 0.939, *p* < 0.0001); and rear hook *r*_*s*_(10) = 0.976, *p* < 0.0001). There was statistically significant, positive correlations between absolute peak impact force and relative peak impact force in the jab (*r*_*s*_(10) = 0.818, *p* = 0.004); cross (*r*_*s*_(10) = 0.809, *p* = 0.005); lead hook (*r*_*s*_(10) = 0.709 *p* = 0.022); and rear hook (*r*_*s*_(10) = 0.936 *p* < 0.0001). There was also statistically significant, positive correlation between relative peak impact force and RFD in the jab (*r*_*s*_(10) = 0.830, *p* = 0.003); cross (*r*_*s*_(10) = 0.721, *p* = 0.019); lead hook (*r*_*s*_(10) = 0.721, *p* = 0.019); and rear hook (*r*_*s*_(10) = 0.948, *p* < 0.0001.

### Punching trials

Table 1 highlights the mean ± SD of peak punch impact force and RFD data from all maximal punch trials. When averaged across four repetitions from both trials, boxers in the present study produced the greatest peak absolute and relative impact forces in the rear hook 2624 ± 581 N, 33.1 ± 4.3 N.kg^-1^, followed by the lead hook 2524 ± 532 N, 31.8 ± 3.7 N.kg^-1^, cross 2425 ± 545 N, 30.6 ± 3.8 N.kg^-1^, and the jab 1645 ± 537 N, 20.6 ± 4.8 N.kg^-1^. Rate of force development followed the same trend 296448 ± 101823 N.s^-1^; 256813 ± 81735 N.s^-1^; 193004 ± 62671; 116675 ± 41577 N.s^-1^ for the rear hook, lead hook, cross, and jab, respectively.

## Discussion

The aims of this study were to examine the test-retest reliability of senior elite amateur boxer’s punch force variables, as quantified by a vertically mounted force plate, and to analyse and report said punch force capabilities. The findings suggest absolute peak impact force and RFD in all punches represent excellent levels of within-day reliability, and good to excellent levels of between-day reliability (ICC 0.89–0.99). The vertically mounted force plate was sensitive to change for most variables, whereby the SEM was typically similar to, or slightly greater than the SWC _(0.2)_, yet smaller than the SWC _(0.6)_. Both relative peak punch force and RFD were strongly correlated with absolute peak punch force (*r* = 0.818–0.745), in agreement with previous research [1]. More specifically, the findings of the current study may agree with a suggestion by Dunn et al., [1], that absolute RFD may show stronger correlations with peak punch force, in contrast to relative RFD (i.e., RFD at select time. However, whist stronger relationships were found in absolute RFD in the current study compared to relative markers of RFD in the aforementioned study [1], it is important to note that relative RFD was not explored in the current study.

Regarding force production capabilities, when averaged across all trials, amateur boxers were able to produce ~2624 N of force in a rear hook punch, corresponding to a relative force of ~ 33 N.kg^-1^. Likewise, when averaged across all trials, amateur boxers exhibited an RFD of 296448 N.^s-1^.

### Reliability and sensitivity

There is only limited research on the reliability of punch force variables in amateur boxing [1]. In the present study, ICC scores on both day 1 and day 2 ranged from 0.87 to 0.99 The greatest ICC’s were found in the absolute peak impact force across day 1 and day 2 testing sessions (0.96–0.99), representing excellent within-day reliability, though this varied across punches. This variation across punches is also evident in the findings of Dunn et al., [1]. Weir proposed that ICC values can be dependent on between-subjects variability [10], and that other measures of reliability should also be explored. Therefore, the current study also included measures of absolute reliability (CV%, SEM). The CV% on both day 1 and day 2 range from 3–10% in all punch force variables. The SEM for peak punch force across all punches on day 1 and day 2, ranged from 67 to 147. This fell within the ranges found in previous research [1]. Our findings show that for absolute peak impact forces, SEM was most frequently greater than the SWC _(0.2)_ but always less than the SWC _(0.6)_, in agreement with previous work [1]. Further, day 1 cross and rear hook, and day 2 jab and lead hook SEM was less than the SWC _(0.2)_. This suggests that the vertically mounted force plate can be considered useful in detecting small to moderate practical changes in amateur boxer’s absolute peak punch force. Relative force showed good to excellent reliability (ICC 0.87–0.95) across all punches on day 1 and day 2, though this was slightly lower than that observed in absolute peak force, in accordance with previous literature [1]. In relation to the SEM, this was consistently greater than the SWC _(0.2)_ but less than the SWC _(0.6)_. Likewise, RFD showed excellent reliability (ICC 0.93–0.99) across all punches on day 1 and day 2. In relation to the SEM, this was consistently greater than the SWC _(0.2)_, but less than the SWC _(0.6)_, with the exception of the lead hook (< SWC _0.2_). When comparing within-day and between-day ICC’s, the majority of variables were similar, with a few notable exceptions. Between-day ICC’s for absolute and relative peak force in the lead hook (ICC 0.91, 0.58) and rear hook (ICC 0.89, 0.61) were lower when compared to within-day values; however, these were still classed as good to excellent, and moderate ICC for absolute peak force, and relative peak force, respectively. The SEM of most variables were greater than SWC _(0.2)_ but less than SWC _(0.6)_. The above data suggests that the vertically mounted force plate may be both reliable, and a useful tool in detecting small to moderate practical changes in punch performance.

### Punch performance

When averaged across all trials, the jab produced the lowest absolute and relative peak force (1645 ± 537 N, 20.6 ± 4.8 N.kg^-1^), and RFD (116675 ± 41577 N.s^-1^) of all punches in the present study. When compared to other research, the absolute peak force is similar to that of elite English amateurs [16], but markedly different to other studies of a similar experimental design [1, 3, 14]. One potential reason for this, is differences in the execution of the jab between-studies. For example, previous research [1] has reported much lower absolute peak forces in the jab where this was part of combination punching (i.e., setting up a more forceful punch), and not isolated maximal efforts such as in the present study. Dunn et al., [1] noted the highest CV’s were found in the jab on day 2 (4.4–13.6%), and attributed this to potential variation in technique, as described above. The distance at which a boxer performs a jab may also influence the force produced [3], and this may be dependent on whether the jab is an isolated punch, or as part of a combination. Another potential reason for between-study differences in performance of the jab, and indeed all punches, may be due to variations in participant skill level.

The jab has the shortest delivery time of all punch types, likely due to the shortest trajectory and deviation from stance [30]. The jab may therefore be considered the punch with the least risk of a counter-attack, which may also, in addition to the previous point, partly explain the fact that it is the most frequently performed punch in amateur boxing [31, 32]. A classic study by Filimonov and colleagues [33] first presented the notion that the lower extremities provide a large contribution to punching forces, although it was unclear how the authors reached this conclusion. Stanley et al., [30] found that the jab exhibited relatively lower peak lead leg ground reaction forces (GRF) when compared to other punches, and that the jab may also be less reliant on trunk rotation. It is therefore unsurprising that the jab punch produced the lowest absolute and relative peak force of all punch types in the present study. Compared to punch force, RFD has received much less focus in the literature, despite being a critical factor to effective punch performance [2]. More specifically, boxers have an extremely small amount of time to deliver a punch, with research by Stanley et al., [30] showing that this may range from 405ms to 657ms across maximal punches. Thus, being able to produce large amounts of force, quickly, is desirable for the boxer [34]. When averaged across all trials, boxers produced the lowest RFD in the jab (116675 ± 41577 N.s^-1^), though this may be expected, due to the jab typically being a ‘set-up’ punch, as mentioned previously.

In agreement with previous research [3, 14–16], amateur boxers in the present study produced greater absolute and relative peak forces in the rear cross (2425 ± 545 N, 30.6 ± 3.8 N.kg^-1^) when compared to the jab. The rear cross technique comprises a more linear muscle recruitment pattern when compared to the jab, starting at the lower extremities, travelling through to the upper extremities, with particular emphasis on rotation at the trunk [35]. More specifically, this kinetic sequence starts with the production of large GRF, rear leg drive and transfer of bodyweight from rear foot to front foot, rotation at the pelvis and trunk, and the propulsion of the upper extremities at high velocity before impact [4, 30, 35]. In the punch force literature, the cross frequently produces the highest peak forces; however, this may be due to several studies not including data on hooks and uppercuts. The absolute peak impact force reported in the present study, was similar to that produced by England senior international boxers (2643 ± 1273 N) [16], yet different to other studies. For example, Waliko et al., [36] and Smith et al., [14] both reported much higher absolute peak forces (3427 N; 3722–4800 N) in the cross when compared to the present study; however, these studies included Olympic level boxers and those with international competition experience. Whilst the present study included boxers on national squads, and several national champions and finalists, classed as senior elite boxers, Olympic boxing represents the true pinnacle of amateur boxing. Indeed, when looking more closely at the individual data in the present study, it was clear that boxers who had been selected to represent their nation at international competition, exhibited closer values to the aforementioned literature. Once again, it is worth noting the varied methods of punch force quantification between studies. Walilko et al., [36] utilised load cells and a tekscan pressure sensor within a hybrid dummy, in addition to accelerometers placed in the boxers hands, whilst Smith et al., [14] used wall-mounted piezoelectric force transducers. When averaged across all trials, RFD in the cross was 193004 ± 62671 N.s^-1^. The only other study to assess RFD in the rear cross in boxing from Crouch et al., [37] currently in the form of a research abstract, though the markedly lower RFD (range ~ 88000–158000 N.S^-1^) may reflect the novice level of participants in that study.

Boxers in the present study produced the greatest absolute and relative peak impact forces in the rear hook (2624 ± 581 N, 33.1 ± 4.3 N//kg), followed by the lead hook (2524 ± 532 N, 31.8 ± 3.7 N.kg^-1^), with values similar to that generated by Australian and English national level boxers [16, 17]. The hook technique shares some similarities with the cross, in that it also comprises considerable peak lead leg GRF (where force is transferred from rear to front foot), rotation at the trunk, and high velocities of the most distal point towards the target [1, 14, 30, 38]. The hook comprises a sweeping motion throughout a greater trajectory than other punches, whereby the shoulder abducts to an angle of ~ 90° to the torso [23, 30]. This technique includes a stretch-shortening cycle (SSC) component whereby a pre-stretch of trunk and upper body musculature is performed before propelling the upper extremities at high velocities towards the target. Stanley et al., [30] notes that a key difference in hooks, compared to a cross, is the fixed elbow position in the latter, as opposed to the rapid elbow extension in the former. Further, the peak elbow joint angular velocity before the elbow’s ~90° position, and the angular velocities generated at the shoulder during this sweeping motion across a longer trajectory than the cross, rapidly accelerates the first towards the target [30, 38]. This may somewhat explain the greater absolute and relative peak impact forces observed in the hooks in the present study. The tendency for rear hooks to produce more force when compared to lead hooks, may be a product of the greater GRF production [30], thus greater kinetic energy produced and transferred throughout the kinetic chain. Dunn et al., [1] also postulates that this may be explained by the greater rotation at the trunk and centre of mass movement in the rear hook punch. The above biomechanical factors may also explain the greatest RFD being found in the rear hook (296448 ± 101823 N.s^-1^) followed by the lead hook 256813 ± 81735 N.s^-1^ in the present study. However, once again, the lack of previous literature reporting this variable means comparisons are not possible.

## Strengths and limitations

The main strength of the current study is that it provides valuable data on the within and between-day reliability and sensitivity of a method of punch force quantification. Specifically, the use of a force plate beyond its typical application, and within a PAPE study, is a novel approach. Coaches may utilise vertically mounted force plates to monitor changes in punch force or RFD, two key components to an effective punch [1]. Indeed, this study is the first to report RFD values for each punch type, in boxing. There are several potential limitations that the authors wish to note. A potential limitation may be that RFD at select time-points of a punch was not considered. The present study comprised a small sample size, which may result in increased potential for between-individual differences to influence the overall reliability. However, in recruiting 10 senior elite amateur boxers as participants, and assessing their performance via multiple repetitions, across consecutive days, the authors have attempted to circumvent this. It is important to note a limitation of performing maximal punches to a force plate. Whilst a force plate may be a gold standard in measuring force, the protective padding used to attenuate impact forces in the study, thus protect from injury, may have resulted in slightly underestimated force readings in the absence of correcting for this factor. This is common within the scientific literature, and as such, it is somewhat difficult to compare punching forces between studies. However, prior research has suggested that if the tool used to quantify striking force is reliable, and sensitive to change, then this is still a valuable tool for the researcher, coach, and practitioner in monitoring changes in punch performance in trained combat athletes [19].

## Conclusion

The vertically mounted force plate used in the present study is a reliable, and sensitive tool in quantifying the punch performance of senior elite amateur boxers. With regard to punch force capabilities, the rear hook was the most forceful punch performed, with senior amateur boxers in the present study producing an absolute and relative force of ~ 2624 N and ~ 33 N.kg^-1^, respectively. This highlights the considerable amount of force that can be produced by the senior elite amateur boxer. Likewise, the same punch showed an absolute RFD of ~296448 N.^s-1^, highlighting the rapid nature of force production involved in maximal punching performance.

From a practical standpoint, the coach or practitioner could confidently utilise the vertically mounted force plate to assess the force production capabilities of boxers, or to assess the efficacy of acute or longer-term training interventions.
